# The Role of Interleukin-18 in Serum and Follicular Fluid during In Vitro Fertilization and Intracytoplasmic Sperm Injection 

**DOI:** 10.1155/2016/6379850

**Published:** 2016-09-22

**Authors:** Veronika Günther, Ibrahim Alkatout, Corinna Fuhs, Ali Salmassi, Liselotte Mettler, Jürgen Hedderich, Nicolai Maass, Mohamed Elessawy, Andreas Gerd Schmutzler, Christel Eckmann-Scholz

**Affiliations:** ^1^Department of Obstetrics and Gynecology, University Hospitals Schleswig-Holstein, Kiel Campus, Arnold-Heller Str. 3, House 24, 24105 Kiel, Germany; ^2^Clinic for Obstetrics and Gynecology Leer, Augustenstr. 35–37, 26789 Leer, Germany; ^3^Institute for Medical Informatics and Statistics, University Hospitals Schleswig-Holstein, Kiel Campus, Brunswiker Str. 10, 24105 Kiel, Germany

## Abstract

Cytokines are key modulators of the immune system and play an important role in the ovarian cycle. IL-18 levels in serum and follicular fluid were analyzed in women undergoing in vitro fertilization (IVF) or intracytoplasmic sperm injection (ICSI) treatment. The cohort study group consisted of 90 women, who were undergoing IVF or ICSI. The body mass index (BMI) was determined in all patients; IL-18 levels were measured in follicular fluid and serum. IL-18 levels in serum were significantly higher than those in follicular fluid. The median level in serum was 162.75 (80.21) pg/mL and that in follicular fluid, 138.24 (91.78) pg/mL. Women undergoing IVF treatment had lower IL-18 levels in serum (median, 151.19 (90.73) pg/mL) than those treated with ICSI (median, 163.57 (89.97) pg/mL). The correlation between IL-18 levels in serum and BMI was statistically significant, as well as the correlation between IL-18 levels in follicular fluid and ovarian stimulation response (*p* = 0.003). IL-18 was correlated with the response to ovarian stimulation and was the reason for successful pregnancy after IVF or ICSI treatment. Among other cytokines, IL-18 appears to be a promising prognostic marker of success in reproductive treatment and should be evaluated as such in further prospective studies.

## 1. Introduction

Understanding pre- and periconception processes is essential for improving success rates in reproductive medicine. Mere 20–25% of transferred embryos are eventually born [1]. The implantation depends on a receptive endometrium, a normal blastocyst, and synchronized maternal-fetal interaction. A cascade of cytokines, chemokines, and growth factors mediate the interaction before fertilization of the oocyte and its implantation in the endometrium [2–4]. To accomplish fertilization, an oocyte of good quality must meet a sperm with low DNA damage. It then becomes a blastocyst, which is able to communicate with the maternal endometrium. Pregnancy occurs only in the presence of immunological tolerance and a functioning endocrine and immune system. Cytokines, originally identified as products of immune cells, are important mediators of immune response. These proteins are able to stimulate or inhibit cell growth, regulate cell differentiation, induce cell chemotaxis, and modulate the expression of other cytokines [5].

Interleukin-18 (IL-18) is a proinflammatory cytokine starting the cascade of additional inflammatory cytokines. It was discovered quite recently and was initially described as an interferon-gamma (IFN^*γ*^) inducing factor [6]. IL-18 can stimulate responses mediated by T-helper type 1 (T_H_1) and T-helper type 2 (T_H_2) [7, 8] and is part of the IL-1 family because of its structural homology, receptor family, intracellular transduction pathway, and biological effects [7, 9]. The initially inactive precursor of IL-18 is pro-IL-18, which is cleaved by IL-1*β*-converting enzyme/caspase-1 [7]. IL-18 triggers activation of the transcription factor “nuclear factor kappa-light-chain-enhancer of activated B-cells” (NF-kB) and generates the cascade of proinflammatory cytokines including TNF*α*, IL-1*β*, IL-6, and IL-2 receptor. Further chemokines such as IL-8 and macrophage inflammatory protein 1 alpha (MIP-1*α*) and adhesion molecules such as ICAM-1 are also upregulated [7, 10]. Skurk et al. pointed out that IL-18 is secreted from adipocytes: IL-18 levels are high in obese women but decrease after weight loss [11, 12]. Cytokines are synthesized by a wide range of nonimmune cell types, including normal ovarian cells [13]. Cytokine function in the ovary includes the provision of follicular growth, steroidogenesis, activation of leukocytes necessary for ovulation, and tissue changes during ovulation, luteinization, and luteolysis [5, 14].

The aim of the present study was to determine IL-18 levels in follicular fluid and serum during in vitro fertilization (IVF) on the one hand and during intracytoplasmic sperm injection (ICSI) on the other of infertile women undergoing reproductive measures and their correlation with the outcome of pregnancy. The effect of follicular fluid and serum IL-18 levels on the outcome of pregnancy, obesity, and the number of acquired oocytes was also analyzed. Follicular fluid was obtained from all mature follicles during the oocyte pick-up procedure.

## 2. Materials and Methods

The study group consisted of 90 women aged on average 34 years (range, 25–43 years) who were undergoing IVF or ICSI treatment at the Division of Reproductive Medicine, Department of Obstetrics and Gynecology, University Hospitals Schleswig-Holstein, Kiel Campus. Eighteen women underwent IVF treatment and 72 underwent ICSI treatment from 2012 to 2013. The body mass index (BMI) was determined in all patients.  Table 1 summarizes these data mentioned before. The local ethics committee for human research approved the study and informed consent was obtained from all patients.

Ovarian hormonal stimulation was conducted according to a protocol of gonadotropin-releasing hormone (GnRH) agonist in order to suppress a spontaneous ovulation: nafarelin acetate (Synerela, Syntex, Germany) 900 mg given daily by the transnasal route or DTRP6L (Decapeptyl, Ferring, Germany) 100–500 *μ*g by the subcutaneous route. Once GnRH agonists had induced a state of hypoestrogenism, exogenous FSH (Gonal-F, Serono, Germany) or human menopausal gonadotropin (hMG) (Menogon, Ferring, Germany) or even a combination of both was given to stimulate ovarian follicle development. During the stimulation period, estradiol levels were measured and the follicles visualized by transvaginal ultrasound. Human chorionic gonadotropin (Choragon, Ferring, Germany), 5000–10000 IU, was given as an intramuscular injection to trigger oocyte release. The oocyte pick-up was scheduled 36 hours later, even before ovulation. The measurement of the hormone concentrations mentioned above was performed using ELISA.

Ovarian stimulation response was divided into two groups: a gain of < 6 oocytes was defined as a weak response, whereas a gain of ≥ 6 oocytes was defined as a good response.

After recovery, oocytes were washed free of the follicular fluid. The maturity of the oocyte was assessed after mechanical dissection of cumulus oophorus and corona radiata. Oocytes were incubated for 4 hours in IVF medium with 5.0 mg/mL of human serum albumin (HSA) (SAGE In Vitro Fertilization, Trumbull, CT, USA) at 37°C in 5.8% CO_2_ and humidified air. Semen samples were washed by centrifugation at 300 g with Quinn's Sperm Washing Medium (SAGE In Vitro Fertilization) and subsequently processed by the swim-up method [15] in the IVF medium with 5.0 mg/mL of HSA (SAGE In Vitro Fertilization). Each oocyte was inseminated with 40–80 × 10^3^ of motile sperm. Fertilization was monitored for about 20 h after insemination, and zygotes were placed into the IVF cleavage medium with 5.0 mg/mL of HSA (SAGE In Vitro Fertilization). The zygote was cultured for 2–6 days in the above-described growth medium and was then implanted in the uterus (IVF). In most situations, such as low sperm count or motility, a single sperm was injected directly into the egg using intracytoplasmic sperm injection (ICSI). For the ICSI procedure, 1-2 *μ*L washed spermatozoa were placed in 7% polyvinylpyrrolidone (PVP, SAGE IVF) and the sperm was injected using standard techniques [16]. Each embryo was cultured in a single drop of 20 *μ*L medium (Quinn's Advantage, cleavage medium; SAGE IVF) supplemented with 10% synthetic serum substitute (Quinn's Advantage Serum Protein Substitute; SAGE IVF) that was covered with mineral oil (SAGE IVF) in an atmosphere of 5% O_2_, 5% CO_2_, and 90% N_2_ at 37°C.

Embryos were examined for their quality three days after IVF or ICSI. They were classified according to blastomere number and fragmentation and divided into good and poor embryos.

Preovulatory follicular fluid (FF) was collected from all picked-up oocytes. Blood samples were taken the same day as oocyte retrieval and centrifuged at 350 g for 10 minutes. The supernatant was separated and stored at −80°C. IL-18 levels in serum and follicular fluid were quantified using the Human IL-18 ELISA Kit (MBL, Japan; Code number 7620).

SPSS (number 20) was used for statistical analysis and to perform the Mann–Whitney *U* test and Kruskal-Wallis test. Pearson's correlation coefficient was used to determine correlations. The level of statistical significance was set to *p* ≤ 0.05. Receiver Operating Characteristic (ROC) curves were used to confirm statistical significance with the optimal cut-off values determined by the software. The results are presented as median and interquartile range (IQR). The outliers are marked with a circle and the extreme values with an asterisk in the statistical analyses, but they were excluded from the statistical tests.

## 3. Results

### 3.1. IL-18 Levels in Serum and Follicular Fluid

**Figure 1:**
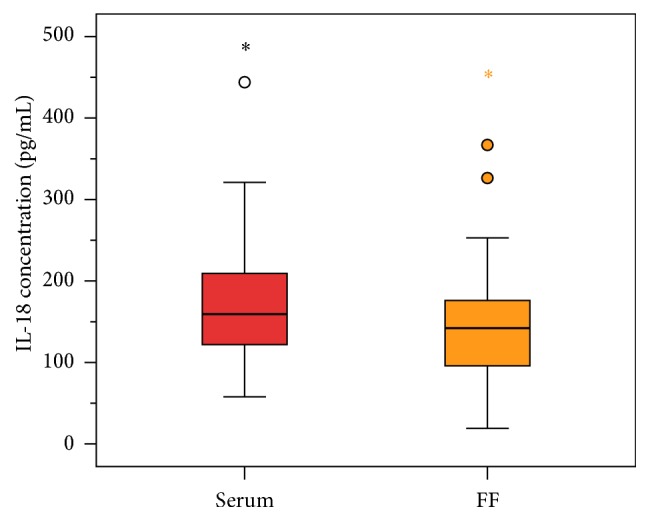
IL-18 levels in serum and follicular fluid (FF). The *x*-axis shows the respective measures for serum and follicular fluid and the *y*-axis the IL-18 concentration (pg/mL). IL-18 levels were significantly higher in serum than in follicular fluid. The median level in serum was 162.75 (80.21) pg/mL and that in follicular fluid, 138.24 (91.78) pg/mL (*p* < 0.0001, Wilcoxon's test).

IL-18 levels, measured in 90 patients, were significantly higher in serum than in follicular fluid. The median level in serum was 162.75 (80.21) pg/mL and that in follicular fluid, 138.24 (91.78) pg/mL (*p* < 0.0001, Wilcoxon's test, Figure 1).

### 3.2. IL-18 Levels in IVF versus ICSI

**Figure 2:**
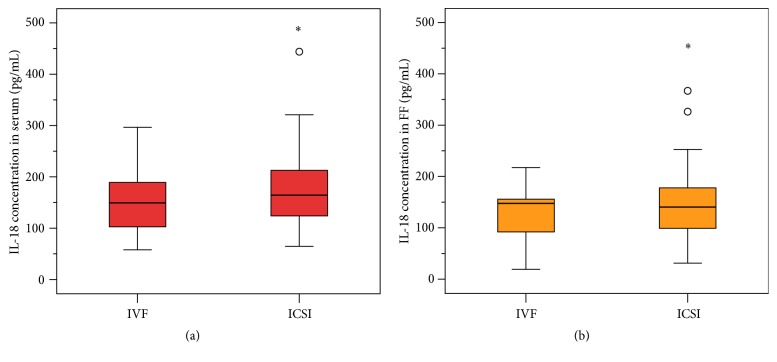
(a) and (b) IL-18 levels in serum (a) and follicular fluid (FF) (b), depending on whether IVF or ICSI was performed. The *x*-axis shows the performed reproductive method (IVF versus ICSI) and the *y*-axis shows the IL-18 concentration (pg/mL) in serum (a) and in follicular fluid (b). Women undergoing IVF treatment had lower IL-18 levels in serum (median, 151.19 (90.73) pg/mL) than those treated with ICSI (median, 163.57 (89.97) pg/mL). These differences were not statistically significant according to the Mann–Whitney *U* test (*p* = 0.265). IL-18 levels in follicular fluid were also lower after IVF treatment (median, 152.09 (78.51)) than they were after ICSI (median 154.12 (84.18) pg/mL); the differences were also not significant according to the Mann–Whitney *U* test (*p* = 0.483).

Eighteen patients underwent IVF treatment and 72 underwent ICSI treatment. Figures 2(a) and 2(b) show IL-18 levels in serum and follicular fluid, depending on whether IVF or ICSI was performed. Women undergoing IVF treatment had lower IL-18 levels in serum (median, 151.19 (90.73) pg/mL) than those treated with ICSI (median, 163.57 (89.97) pg/mL). These differences were not statistically significant according to the Mann–Whitney *U* test (*p* = 0.265). IL-18 levels in follicular fluid were also lower after IVF treatment (median, 152.09 (78.51)) than they were after ICSI (median 154.12 (84.18) pg/mL); the differences were also not significant according to the Mann–Whitney *U* test (*p* = 0.483).

### 3.3. IL-18 Levels in Serum and Follicular Fluid in Relation to Pregnancy

Twenty-eight of 90 women were pregnant after completing IVF or ICSI treatment, which equals a rate of 31.1%. The difference between pregnant and nonpregnant women with regard to IL-18 levels in serum and follicular fluid was not significant according to the Mann–Whitney *U* test (IL-18 in serum *p* = 0.542; IL-18 in follicular fluid; *p* = 0.379).

### 3.4. The Effect of BMI on IL-18 Levels in Serum and Follicular Fluid (FF)

**Figure 3:**
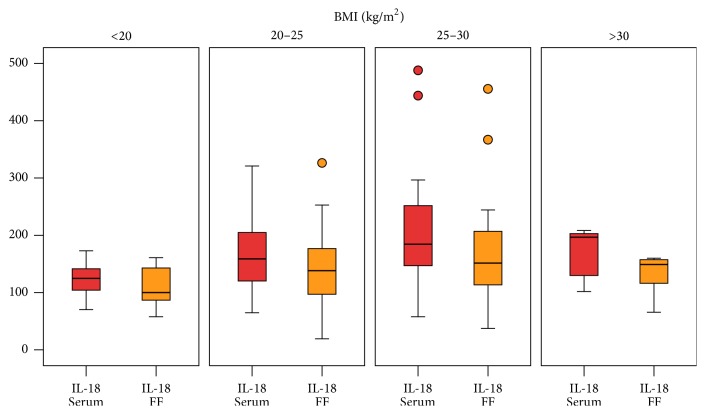
The effect of BMI on IL-18 levels in serum and follicular fluid (FF). The *x*-axis shows the categorization of the patients concerning the BMI. They were divided into four groups (group 1: BMI < 20 (*n* = 11), group 2: BMI 20–25 (*n* = 48), group 3: BMI 25–30 (*n* = 23), and group 4: BMI > 30 (*n* = 5)). The *y*-axis shows the IL-18 concentration (pg/mL) in serum and in follicular fluid. The IL-18 levels in serum increased with a rising BMI (group 1: median 124.78 (40.40) pg/mL, group 2: median 158.91 (86.66) pg/mL, group 3: median 184.54 (127.69) pg/mL, and group 4: median 196.69 (89.72) pg/mL). Based on the Kruskal-Wallis test, the correlation between IL-18 levels in serum and BMI was statistically significant (*p* = 0.027). IL-18 levels in follicular fluid also increased with a rising BMI (group 1: median 100.15 (63.33) pg/mL, group 2: median 138.23 (81.01) pg/mL, group 3: median 151.47 (115.07) pg/mL, and group 4: median 149.00 (67.68) pg/mL). However, the relationship between IL-18 levels and BMI was not significant according to the Kruskal-Wallis test (*p* = 0.071).

Depending on their BMI, the patients were divided into four groups (group 1: BMI < 20 (*n* = 11), group 2: BMI 20–25 (*n* = 48), group 3: BMI 25–30 (*n* = 23), and group 4: BMI > 30 (*n* = 5)). IL-18 levels in serum increased with a rising BMI (group 1: median 124.78 (40.40) pg/mL, group 2: median 158.91 (86.66) pg/mL, group 3: median 184.54 (127.69) pg/mL, and group 4: median 196.69 (89.72) pg/mL), Figure 3. Based on the Kruskal-Wallis test, the correlation between IL-18 levels in serum and BMI was statistically significant (*p* = 0.027).

IL-18 levels in follicular fluid also increased with a rising BMI (group 1: median 100.15 (63.33) pg/mL, group 2: median 138.23 (81.01) pg/mL, group 3: median 151.47 (115.07) pg/mL, and group 4: median 149.00 (67.68) pg/mL), Figure 3. However, the relationship between IL-18 levels and BMI was not significant according to the Kruskal-Wallis test (*p* = 0.071).

### 3.5. The Effect of Gained Oocytes on IL-18 Levels in Serum and Follicular Fluid (FF)

**Figure 4:**
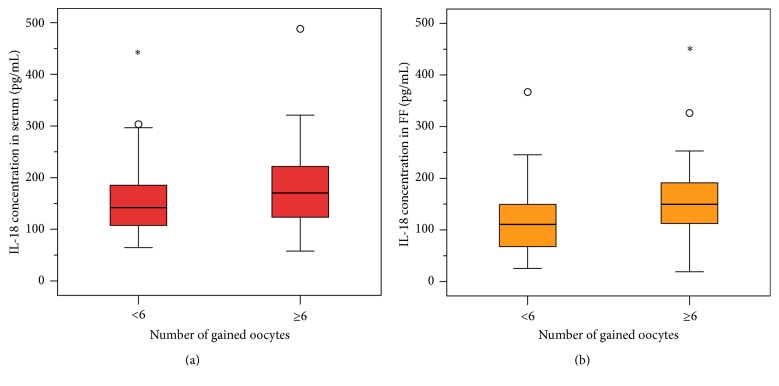
(a) and (b) The effect of gained oocytes on IL-18 levels in serum and follicular fluid (FF). The *x*-axis shows the number of gained oocytes (<6 versus ≥6) and the *y*-axis shows the IL-18 concentration (pg/mL) in serum (a) and in follicular fluid (b). A gain of < 6 oocytes was defined as a weak response (*n* = 28); a gain of ≥ 6 oocytes was defined as a good response (*n* = 62). IL-18 levels in serum (group 1: median 153.47 (75.6) pg/mL, group 2: median 169.21 (95.71) pg/mL) as well as in follicular fluid (group 1: median 117.18 (87.98) pg/mL, group 2: median 155.74 (66.35) pg/mL) increased from group 1 to group 2 (a, b). The correlation between IL-18 levels in serum and the ovarian stimulation response was not statistically significant (*p* = 0.116), whereas the correlation between IL-18 levels in follicular fluid and the ovarian stimulation response was significant (*p* = 0.003), according to the Mann–Whitney *U* test.

With regard to their ovarian stimulation response the women were divided into two groups: group 1: a gain of < 6 oocytes was defined as a weak response (*n* = 28); group 2: a gain of ≥ 6 oocytes was defined as a good response (*n* = 62). IL-18 levels in serum (group 1: median 153.47 (75.6) pg/mL, group 2: median 169.21 (95.71) pg/mL) as well as in follicular fluid (group 1: median 117.18 (87.98) pg/mL, group 2: median 155.74 (66.35) pg/mL) increased from group 1 to group 2 (Figures 4(a) and 4(b)). The correlation between IL-18 levels in serum and the ovarian stimulation response was not statistically significant (*p* = 0.116), whereas the correlation between IL-18 levels in follicular fluid and the ovarian stimulation response was significant (*p* = 0.003), according to the Mann–Whitney*U* test. We performed the Spearman correlation between IL-18 follicular levels/IL-18 in serum and the number of oocytes retrieved. There is no correlation between IL-18 in serum and retrieved oocytes (*R* = 0.076; *p* = 0.478), but there is a positive correlation between IL-18 in follicular fluid and retrieved oocytes (*R* = 0.233; *p* = 0.027).

### 3.6. Receiver Operating Characteristic (ROC) Analysis

**Figure 5:**
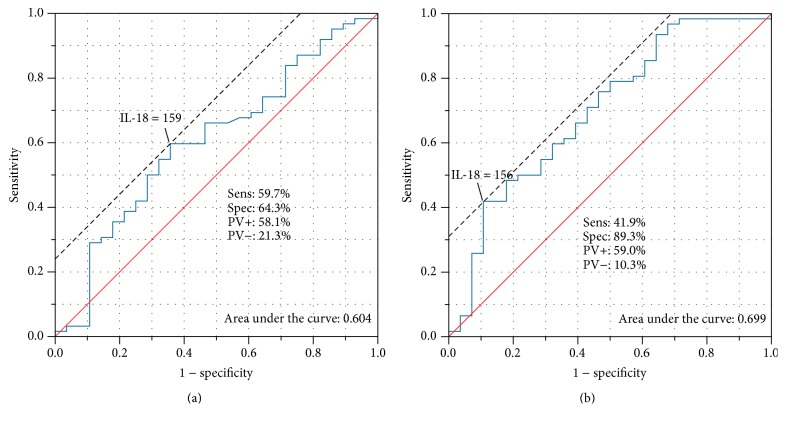
(a) and (b) Area under the curve (AUC): correlation between IL-18 levels in serum (a) and follicular fluid (FF) (b) and the strength of the relationship concerning the response to ovarian stimulation. A cut-off value in serum (a) and FF (b) was determined, and the respective sensitivity and specificity values were calculated. PV+: positive predictive value; PV−: negative predictive value.

An ROC analysis was performed to establish the strength of the relationship between IL-18 levels in serum and follicular fluid and the response to ovarian stimulation (measured as the number of gained oocytes). Sensitivity and specificity were determined using a cut-off value. Thus, ROC analysis yielded an AUC (area under the curve) value of 0.604 concerning IL-18 levels in serum and the ovarian response (Figure 5(a)). The AUC value for IL-18 levels in follicular fluid and the ovarian response was 0.699 (Figure 5(b)). At a cut-off value of 158.63 pg/mL in serum, the sensitivity was 59.7% and the specificity 64.3% (Figure 5(a)). The cut-off value was set to 155.96 pg/mL for IL-18 levels in follicular fluid. This resulted in a sensitivity of 41.9% and a specificity of 89.3% (Figure 5(b)). In summary, there is a stronger correlation between ovarian stimulation response and IL-18 levels in follicular fluid than in serum, which supports the above mentioned data.

## 4. Discussion

We evaluated IL-18 levels in the serum and follicular fluid of infertile women undergoing IVF or ICSI. IL-18 levels in serum were significantly higher than those in follicular fluid (*p* < 0.0001). Gutman et al. [17] also analyzed IL-18 levels in serum and preovulatory follicular fluid during IVF and noted higher levels in serum (367.54 (196.45) pg/mL) than in follicular fluid (216.82 (185.74) pg/mL; *p* < 0.0001). Besides, they observed a positive correlation between follicular fluid levels of IL-18 and the number of retrieved oocytes (*p* = 0.019).

The presence of IL-18 in follicular fluid suggests ovarian secretion. Espey compared ovulation with an inflammatory reaction, involving neutrophil granulocytes, bradykinin, histamine, and cytokines [18]. A number of morphological changes occur at the apex of a follicle wall during ovulation. Further studies have shown that the tenacious connective tissue layers of the tunica albuginea and the theca externa must be weakened before the follicle wall can dissociate and break open under the force of modest intrafollicular pressure [19].

In our study, 5,000–10,000 IU of human chorionic gonadotropin (Choragon, Ferring, Germany) was given as an intramuscular injection to trigger oocyte release. The oocyte pick-up was scheduled 36 hours later, even before ovulation. Owing to this specific chronological arrangement, the inflammatory reaction even before ovulation seems to trigger the production of IL-18. We also noted a significant correlation between IL-18 levels in follicular fluid and a high response to ovarian stimulation (≥6 oocytes). By contrast, the correlation between IL-18 levels in serum and the ovarian stimulation response was not statistically significant. IL-18 seems to have a role in oocyte release, but it is not important for folliculogenesis. Further studies have addressed hormonal changes during ovulation and their benefits in natural family planning. Analogous to the ferning pattern in the cervical mucus, a woman's saliva changes during the menstrual cycle. Ferning is caused by NaCl, which increases cyclically under the influence of estrogen and coincides with the woman's fertile period. A drop of saliva from below the tongue is placed on the lens of a microscope (e.g.,* Geratherm* ovu control). Ferning is a crystal-like pattern which indicates a fertile period, whereas simple dot patterns and lines indicate no ovulation. Thus, changes in saliva caused by increased estrogen levels can be used to detect ovulation and maximize the likelihood of conception [20].

Several studies have addressed the association between IL-18 levels in serum and polycystic ovaries [21]. Overproduction of proinflammatory factors is associated with obesity and diabetes [22, 23]. IL-18, a member of the IL-1 family, is increased in the presence of obesity, diabetes, and the polycystic ovary syndrome (PCOS). Serum IL-18 levels were elevated in women with PCOS compared to controls (*p* = 0.003) and clearly associated with insulin resistance [24].

Three patients in our study developed an ovarian hyperstimulation syndrome (OHSS) which resulted in large cysts and the accumulation of fluid in the abdomen, along with a high risk of thrombosis. In our study, we classed the patients with OHSS as outliers and excluded them from the statistical tests. The serum IL-18 levels of these patients were significantly higher (598 (182) pg/mL) than those of women without OHSS (324 (248) pg/mL) (*p* = 0.04). Barak et al. analyzed IL-18 concentrations as a marker of OHSS and showed significantly higher IL-18 levels in the serum, peritoneal, and pleural fluid of patients with severe OHSS as compared with two control groups. On transition to the diuretic phase and resolution, serum IL-18 dropped significantly [9].

Twenty-eight of 90 women became pregnant after IVF or ICSI, which is equivalent to a rate of 31.1%. The difference in IL-18 levels in serum and follicular fluid between pregnant and nonpregnant women was not significant. How the maternal immune system adapts to the fetus is not fully understood; alloimmune as well as autoimmune mechanisms with natural killer (NK) cells and autoantibodies appear to be involved in the process. Successful pregnancy is regarded as a manifestation of Th1-Th2 cooperation, with a predominantly Th2-type lymphocyte response involving IL-12, IL-15, IL-18, and further soluble factors dependent on NK cells [25]. Changes in the number of circulating NK may be associated with impaired pregnancy. Inflammatory/autoimmune processes caused by NK may result in immunological infertility [25]. A successful pregnancy is associated with the presence of Th2-type cytokines, whereas miscarriage is associated with the production of Th1-type cytokines [26]. Increased serum levels of the Th1-associated cytokines IFN-gamma, IL-12, and IL-18 were registered in women who miscarried compared to those with a healthy pregnancy. Particularly increased IL-18 levels appeared to be critical in early pregnancy and served as a point of differentiation between healthy pregnancies and those culminating in miscarriage [26–28].

IL-18 concentrations in serum and follicular fluid increased as BMI rose. The correlation in serum was statistically significant (*p* = 0.027) and the correlation in follicular fluid nearly significant (*p* = 0.071). Esposito et al. analyzed serum IL-18 levels in 40 obese women und 40 normal-weight women. They found higher IL-18 levels in the former than in the latter and falling IL-18 levels in women who experienced weight loss [12]. This leads to the conclusion that adipose tissue, among other factors, is responsible for the production of IL-18. Obesity is associated with a high risk of developing atherosclerosis, which may be mediated by the increased secretion of proinflammatory cytokines in adipose tissue [29, 30]. In addition to IL-18, IL-6 and TNF*α* are released also by adipose tissue and may induce endothelial expression of chemokines and adhesion molecules, which play an important role in early stages of the atherogenic process [31, 32]. Further epidemiological studies have reported a high vascular risk in connection with increased levels of IL-6 and TNF*α* [33]. As IL-18 induces the production of TNF*α* which, in turn, supports the synthesis of IL-6, IL-18 might well be responsible for a high risk of cardiovascular disease in obese patients [34].

## 5. Conclusion

IL-18 levels in serum were significantly higher than those in follicular fluid. Women undergoing IVF treatment had lower IL-18 levels in serum than those treated with ICSI. After completion of IVF or ICSI treatment, 28 of 90 women became pregnant. The difference in serum IL-18 levels and follicular fluid IL-18 levels between pregnant and nonpregnant women did not achieve statistical significance. The correlation between IL-18 levels in serum and BMI was statistically significant. IL-18 levels in follicular fluid increased with a rising BMI. The correlation between IL-18 levels in serum and ovarian stimulation response was not statistically significant, whereas the correlation between IL-18 levels in follicular fluid and ovarian stimulation response was significant.

In conclusion, we were able to show that IL-18 is correlated with response to ovarian stimulation and therefore plays an important role for successful pregnancy after IVF or ICSI treatment. Among other cytokines, IL-18 appears to be a promising prognostic marker for success in reproductive treatment and should be evaluated as such in further prospective studies.

## Figures and Tables

**Table 1:** Patient data.

*Age* (years)	25–43 (mean 34)

	*Number of patients* (*n* = 90)

*Reproductive treatment*	
IVF	18
ICSI	72

*BMI (kg/m* ^*2*^)	
<20	12
21–25	55
>26	23

*Ovarian response*	
<6 oocytes	28
≥6 oocytes	62
